# NT3^P75-2^ gene-modified bone mesenchymal stem cells improve neurological function recovery in mouse TBI model

**DOI:** 10.1186/s13287-019-1428-1

**Published:** 2019-10-24

**Authors:** Ke Wu, Dongdong Huang, Can Zhu, Ella A. Kasanga, Ying Zhang, Enxing Yu, Hengli Zhang, Zhihui Ni, Sheng Ye, Chunli Zhang, Jiangnan Hu, Qichuan Zhuge, Jianjing Yang

**Affiliations:** 1 Department of Neurosurgery, Zhejiang Provincial Key Laboratory of Aging and Neurological Disorder Research, The First Affiliated Hospital of Wenzhou Medical University, Wenzhou, 325000 China; 2 Department of Pharmacology and Neuroscience, University of North Texas Health Science Center, Fort Worth, TX 76107 USA; 3 Department of Molecular Biology, University of Texas Southwestern Medical Center, Dallas, TX 75390 USA

**Keywords:** Traumatic brain injury, NT3^P75-2^, Bone marrow-derived mesenchymal stem cells, Neurological function, P75 signal pathway

## Abstract

**Background:**

The attainment of extensive neurological function recovery remains the key challenge for the treatment of traumatic brain injury (TBI). Transplantation of bone marrow-derived mesenchymal stem cells (BMSCs) has been shown to improve neurological function recovery after TBI. However, the survival of BMSCs after transplantation in early-stage TBI is limited, and much is unknown about the mechanisms mediating this neurological function recovery. Secretion of neurotrophic factors, including neurotrophin 3 (NT3), is one of the critical factors mediating BMSC neurological function recovery. Gene mutation of NT3 (NT3^P75-2^) has been shown to enhance the biological function of NT3 via the reduction of the activation of the P75 signal pathway. Thus, we investigated whether NT3^P75-2^ gene-modified BMSCs could enhance the survival of BMSCs and further improve neurological function recovery after TBI.

**Methods:**

The ability of NT3^P75-2^ induction to improve cell growth rate of NSC-34 and PC12 cells in vitro was first determined. BMSCs were then infected with three different lentiviruses (green fluorescent protein (GFP), GFP-NT3, or GFP-NT3^P75-2^), which stably express GFP, GFP-NT3, or GFP-NT3^P75-2^. At 24 h post-TBI induction in mice, GFP-labeled BMSCs were locally transplanted into the lesion site. Immunofluorescence and histopathology were performed at 1, 3, and/or 7 days after transplantation to evaluate the survival of BMSCs as well as the lesion volume. A modified neurological severity scoring system and the rotarod test were chosen to evaluate the functional recovery of the mice. Cell growth rate, glial activation, and signaling pathway analyses were performed to determine the potential mechanisms of NT3^P75-2^ in functional recovery after TBI.

**Results:**

Overall, NT3^P75-2^ improved cell growth rate of NSC-34 and PC12 cells in vitro. In addition, NT3^P75-2^ significantly improved the survival of transplanted BMSCs and neurological function recovery after TBI. Overexpression of NT3^P75-2^ led to a significant reduction in the activation of glial cells, brain water content, and brain lesion volume after TBI. This was associated with a reduced activation of the p75 neurotrophin receptor (P75NTR) and the c-Jun N-terminal kinase (JNK) signal pathway due to the low affinity of NT3^P75-2^ for the receptor.

**Conclusions:**

Taken together, our results demonstrate that administration of NT3^P75-2^ gene-modified BMSCs dramatically improves neurological function recovery after TBI by increasing the survival of BMSCs and ameliorating the inflammatory environment, providing a new promising treatment strategy for TBI.

## Introduction

Traumatic brain injury (TBI) is a serious social problem worldwide [1]. Globally, TBI results in approximately 10 million hospitalizations and/or deaths every year with an estimated number of 57 million people still suffering the consequences of the condition [2, 3]. These high mortality and morbidity rates create a huge burden for not only the patient’s family but also the health care system [4]. The gold standard for the management of TBI is surgical treatment combined with neurological rehabilitation training to improve neurological prognosis [5]. However, this intervention is not sufficient for full neurological function recovery necessitating the need for new and effective treatment strategies.

The application of mature stem cells, such as bone marrow-derived stem cells (BMSCs), for neurological diseases has been widely explored as a promising regenerative therapeutic strategy [6]. Over the past decade, many studies have shown the ability of BMSCs to survive after transplantation in the injury area in mice TBI models [7, 8]. BMSCs increase the synthesis and expression of diverse neurotrophins and synaptic proteins which enhance motor function recovery after TBI [9]. In addition, a recent study also showed that transplantation of BMSCs downregulates BAX and BAD to promote neurological recovery via increasing the expression of glial cell line-derived neurotrophic factor (GDNF) [10], which has been shown to lead to the suppression of apoptosis [11].

Apart from brain-derived neurotrophic factor (BDNF) and nerve growth factor (NGF), neurotrophin 3 (NT3) has also been found to be beneficial in reducing brain damage [12]. Studies have shown that NT3 enhances the neurological functional recovery by regulating both neurite/axon growth and neuronal survival via activating a specific downstream serine threonine kinase (receptor tyrosine kinase (Trk)) [13]. However, NT3 also binds to P75 neurotrophin receptor (P75NTR) leading to apoptosis during development and injury [14, 15]. The P75NTR is a transmembrane glycoprotein member of the tumor necrosis factor receptor (TNFR) superfamily, characterized by four cysteine-rich domains in its extracellular region [16]. This protein can bind with all neurotrophic factors with a similar affinity to regulate multitudinous neurologic functions [17]. Interestingly, Rabizadeh and his colleagues reported that NGF could also activate P75NTR, which is associated with neural cell demise by apoptosis [18]. In line with this study, another research group demonstrated that the inactivation of P75NTR in Schwann cells promotes spinal cord growth and functional recovery after spinal cord injury (SCI) [19]. Usage of a specific gene-mutated form of NT3, NT3^P75-2^, which binds to P75NTR with a lower affinity, leads to enhanced pro-survival properties of transplanted Schwann cells after SCI [20].

Thus, we sought to determine if NT3^P75-2^ could augment the neurological protective effects of NT3 and if NT3^P75-2^ gene-modified BMSCs could significantly improve the neurological function recovery in a TBI mouse model as well as the underlying mechanisms.

## Materials and methods

### Cell culture

HyClone™ Dulbecco’s modified Eagle’s medium (DMEM) (GE Healthcare Life Science) with 10% fetal bovine serum (FBS) (Corning) was used for mouse motor neuron-like hybrid cell line (NSC-34), PC12 cells, HEK293T, and BMSCs culture. The cell lines of NSC-34, PC12, and HEK293T were bought from American Type Culture Collection (ATCC, USA). PC12 cells were incubated by culture medium containing 50 ng/ml NGF (Peprotech, 450-34) for 24 h and keep passage for the following experiment. BMSCs were isolated from the fetuses of SD rats as previously described [21]. All cells were maintained at 37 °C in a humidified incubator with 5% CO_2_ with cell passage performed, when cell density was ~ 90%. Cryopreservation solutions used comprised of complete medium and 10% dimethyl sulfoxide (DMSO).

### Plasmid construction and virus production

We obtained the NT3 and NT3^P75-2^ sequence from the addgene website which was deposited by Pantelis Tsoulfas lab. The detail mutation information was described in their published paper [20]. In this experiment, the pCDH-CMV-Puromycin lentiviral vector was used for the expression of green fluorescent protein (GFP), GFP-NT3, and GFP-NT3^P75-2^. All plasmids were digested and sequenced for confirmation. For lentivirus production, packaging plasmids (pMDL, VSV-G, and pREV) and lentiviral vectors were transfected into HEK293T cells as previously described [22]. The supernatant of HEK293T cells was collected 24 h and 48 h after transfection and filtered with 0.22-μm strainer [23, 24]. Virus-containing culture media were then collected, filtered, and stored at 4 °C.

### Establishment of stable expression in BMSCs

The fresh virus-containing culture media (GFP, GFP-NT3, and GFP-NT3^P75-2^) was applied to infect the BMSCs. Infection efficiency was checked by detecting the GFP fluorescence (1:500, Santa Cruz Biotechnology) via immunofluorescent staining at 3 days post-infection. The infection efficiency (%) was defined as the ratio of GFP-positive cells divided by the total number of cells (DAPI positive) per field. For statistical analysis, three different wells with five random fields per well were evaluated.

### Cell Counting Kit-8 assay

To determine whether the overexpression of NT3^P75-2^ influenced the proliferation of NSC-34, PC12, and BMSC cells, CCK8 assay was performed. Briefly, after the NSC-34, PC12, and BMSC cells were infected with lentivirus (GFP, GFP-NT3, and GFP-NT3^P75-2^), cells were seeded in 96-well culture plate (1 × 10^4^ per well) in 100 μl of 10% FBS DMEM and incubated for 8 h at 1 and/or 3 and/or 7 days post-infection. 10 μl of CCK8 solution (Dojindo, Japan) was then added to each well, and cells were incubated at 37 °C for 3 h. Absorbance was analyzed at 450 nm by Spectramax 190 (Molecular Devices, USA). Cell growth rate was calculated based on the following equation:

$$ \mathrm{Cell}\ \mathrm{growth}\ \mathrm{rate}\ \left(100\%\right)=\frac{\mathrm{A}\kern0.5em \left(\mathrm{Treatment}\kern0.5em \mathrm{group}\hbox{-} \mathrm{blank}\right)}{\mathrm{B}\ \left(\mathrm{Control}\kern0.5em \mathrm{group}\hbox{-} \mathrm{blank}\right)}\times 100\% $$

HEK293T cell was transfected with plasmids (GFP, GFP-NT3, and GFP-NT3^P75-2^). At 3 days post-transfection, the culture medium was replaced. Twenty-four hours later, the fresh medium was collected and filtered with 0.22-μm strainer and stored at 4 °C. To determine whether the supernatant containing NT3^P75-2^ had the same effect on NSC-34 and PC12 cell growth rate, CCK8 assay was repeated for NSC-34 and PC12 cell lines after treatment with supernatant containing NT3^P75-2^. At 1, 3, and 7 days post-treatment, the medium in every well was replaced and supplemented with 10 μl CCK8 solution to determine the absorbance value just as described earlier.

### TBI mouse model

Nine- to 12-week-old male mice (15–20 g) were housed in a 12-h light-dark cycle with food and water provided ad libitum. Mice were maintained in specific pathogen-free conditions in the Animal Facility at Wenzhou Medical University. The controlled cortical impact (CCI) model was used as previously described [25]. Briefly, the animals were anesthetized using 4% chloral hydrate (10 μl/g, i.p.) and placed in the stereotaxic frame (KOPF, Tujunga, CA, USA). The lesion was created at a velocity of 4 m/s, 2.0 mm depth, and 3 mm diameter piston in the right hemisphere (coordinates from the bregma; AP, − 5 mm; ML + 5 mm) by the Impact One™ Stereotaxic Impactor for CCI (Leica, USA). All mice were randomly divided into five groups: sham group, TBI group, GFP-BMSCs group (mice subjected to TBI and received GFP-BMSC transplantation at 24 h post-TBI), GFP-NT3-BMSCs group (transplanted with GFP-NT3-BMSCs at 24 h post-TBI), and GFP-NT3^P75-2^-BMSCs group (transplanted with GFP-NT3^P75-2^-BMSCs at 24 h post-TBI). All procedures were in compliance with the Animal Care and Use Committee of Wenzhou Medical University.

### BMSC transplantation

BMSCs were digested with 0.25% trypsin-EDTA for 3 min at 37 °C. After which, detached BMSCs were transferred into a centrifuge tube and washed with pre-cold phosphate-buffered saline (PBS) three times. 3 × 10^5^ cells (for each treatment) in 3 μl of DMEM medium were engrafted into the epicenter of the injury site at a delivery rate of 1 μl/min with a Hamilton7000 microinjection needle. Animals in the sham and TBI only groups received saline injections.

### Assessment of neurological function

The modified neurological severity score (mNSS), which includes motor, sensory, reflex, and balance tests, was used for evaluating neurological function. Neurological function grade score ranges from 0 to 18. The higher score, the more severe the neurological impairment. All mice included in this experiment were trained before [26] the induction of TBI. All animals were then evaluated before TBI as well as 3, 7, 14, and 28 days after TBI.

### Assessment of motor function

Motor coordination was assessed using the rotarod test [27]. Mice were trained to perform this test before TBI induction. Briefly, mice were placed on an accelerating rotating rod, which accelerated from 5 to 50 r/min within 5 min. The latency to fall for each mouse was then recorded. An average of three trials was used as a measure of the motor performance of the mice. This test was conducted before TBI and 3, 7, 14, and 28 days after TBI and were scored by blinded experimenters.

### Assessment of brain water content

The brains were harvested at 72 h after TBI. The brains were initially weighed to obtain the wet weight (WW) after which the brains were oven dried at 105 °C for 2 h for the dry weight (DW). The brain water content was calculated according to the following formula:

$$ \mathrm{Brain}\ \mathrm{water}\ \mathrm{content}=\frac{\mathrm{WW}-\mathrm{DW}}{\mathrm{WW}}\times 100\kern0.5em \% $$

### Western blot analysis

This was performed as previously described [28]. Briefly, the different cell samples were lysed with radioimmunoprecipitation assay (RIPA) lysis and extraction buffer (Thermo Fisher, Waltham, MA, USA). The concentration of total protein was determined using the Pierce™ bicinchoninic acid assay (BCA) protein assay kit (Thermo Fisher, Waltham, USA). Proteins were separated by 8–12% SDS-PAGE before transferring to polyvinylidene difluoride (PVDF) membranes (Millipore, Burlington, MA, USA). PVDF membranes were blocked with 3% bovine serum albumin solution (BioFroxx, Germany) and incubated with primary antibodies at 4 °C overnight. The primary antibodies used include neurotrophic tyrosine kinase, receptor, type 3 (TrkC) (rabbit polyclonal, Proteintech, 11999-1-AP, 1:1000), P75NTR (rabbit polyclonal, Proteintech, 55014-1-AP, 1:1000), NT3 (rabbit polyclonal, Proteintech, 18084-1-AP, 1:1000), protein kinase B (AKT) (rabbit polyclonal, Proteintech, 10176-2-AP, 1:1000), phospho-AKT (rabbit polyclonal, Affinity Biosciences, #AF0016, 1:1000), c-Jun N-terminal kinase (JNK) (rabbit polyclonal, Proteintech, 51151-1-AP, 1:1000), phospho-JNK (rabbit polyclonal, Affinity Biosciences, #AF3318, 1:1000), and β-actin (rabbit polyclonal, Abcam, ab8227, 1:1000). PVDF membranes were further incubated with appropriate secondary antibodies (1:10000) for 2 h at room temperature before visualization by WesternBright ECL HRP substrate (Advansta, San Jose, CA, USA).

### mRNA isolation and qPCR

TRIzol reagent (Thermo, 15596018, USA) was used for total RNA isolation, after RNA quantity and quality were identified by NanoDrop ND-1000. Approximately 1 μg of total RNA was converted to the first-strand cDNA using a SuperScript II RT system (Thermo, K1622, USA). qPCR was performed in a 20-μl reaction in the presence of SYBR® Fast qPCR Mix (Takara, RR820A, Japan) on an ABI Q5 Real-Time PCR. The related primers (Sangon Biotech) are listed as follows: TrkC(F): 5′-ATGGAGCTCTACACGGGACT, TrkC(R): 5′-GGTGAGCCGGTTACTTGACA; P75NTR(F): 5′-TGCTGCTGATTCTAGGGATGTC, P75NTR(R): 5′-GGTTCACA CACGGTCTGGT; and gapdh(F): 5′-CTGGCATTGCTCTCAATGACAAC, gapdh(R): 5′-GGTTCACACACGGTCTGGT. The reactions were carried out in triplicate and expressed as the ratio of the cycle threshold value for the cDNA concentration of the target gene normalized to GAPDH using the 2^−ΔΔCt^ method.

### Enzyme-linked immunosorbent assay

To determine the secretion of NT3 or NT3^P75-2^ in NSC-34, PC12, HEK293T cells, or BMSCs after lentivirus infection (NT3 and NT3^P75-2^), the concentrations of NT3 or NT3^P75-2^ from the supernatant of NSC-34, PC12, HEK293T, or BMSCs were measured with the specific NT3 ELISA kits (R&D Systems, MN, USA). For each ELISA analysis, 40 μl of supernatant sample (stock solution) was used in accordance with the manufacturer’s instructions [29].

### Assessment of lesion volume

This was done as previously described [21]. Briefly, mice were sacrificed and transcardially perfused with saline and 4% paraformaldehyde (PFA) 7 days after TBI. The sections were stained by hematoxylin and eosin staining kit (Beijing Solarbio Science & Technology Co., China) and imaged at × 10 magnification with an inverted microscope (Leica Microsystems, Germany). The contralateral and ipsilateral hemisphere volumes were then quantified by ImageJ software (NIH). The lesion volume was calculated using the following equation:

$$ \mathrm{Lesion}\ \mathrm{volume}=\frac{\mathrm{lesion}\kern0.5em \mathrm{volume}}{\mathrm{volume}\kern0.5em \mathrm{of}\kern0.5em \mathrm{contralateral}\kern0.5em \mathrm{hemisphere}}\times \kern0.5em 100\kern0.5em \% $$

### Immunofluorescence assays

Mice (*n* = 4) were sacrificed and transcardially perfused with saline and 4% PFA. Briefly, coronal frozen sections (10 μm) were incubated at 4 °C overnight with the following primary antibodies: anti-ionized calcium-binding adaptor molecule 1 (Iba1) (1:200, Abcam, ab5076), GFP (1:200, Proteintech, 66002-1-Ig), and glial fibrillary acidic protein (GFAP) (1:200, Abcam, ab7260). The sections were then washed three times with PBS with Tween 20 and incubated at room temperature for 1 h with corresponding secondary antibodies (Invitrogen, 1:500). A scanning fluorescence microscope (Leica Microsystems) was used to capture the images of the staining sections. The data were expressed as the average number of positive cells per square millimeter. For statistical analysis, five random images around the TBI area were taken from each slide.

### Statistical analysis

The data were presented as mean ± standard error of mean (SEM) and analyzed by using GraphPad Prism 5 (San Diego, CA, USA). To compare the differences between the two groups, unpaired Student’s *t* test was used. If three or more groups were involved, a one-way or two-way analysis of variance (ANOVA) was utilized. For all comparisons, *p* values less than 0.05 are considered statistically significant.

## Results

### NT3^P75-2^ induction improves cell growth rate of NSC-34 and PC12 cells in vitro

**Fig. 1:**
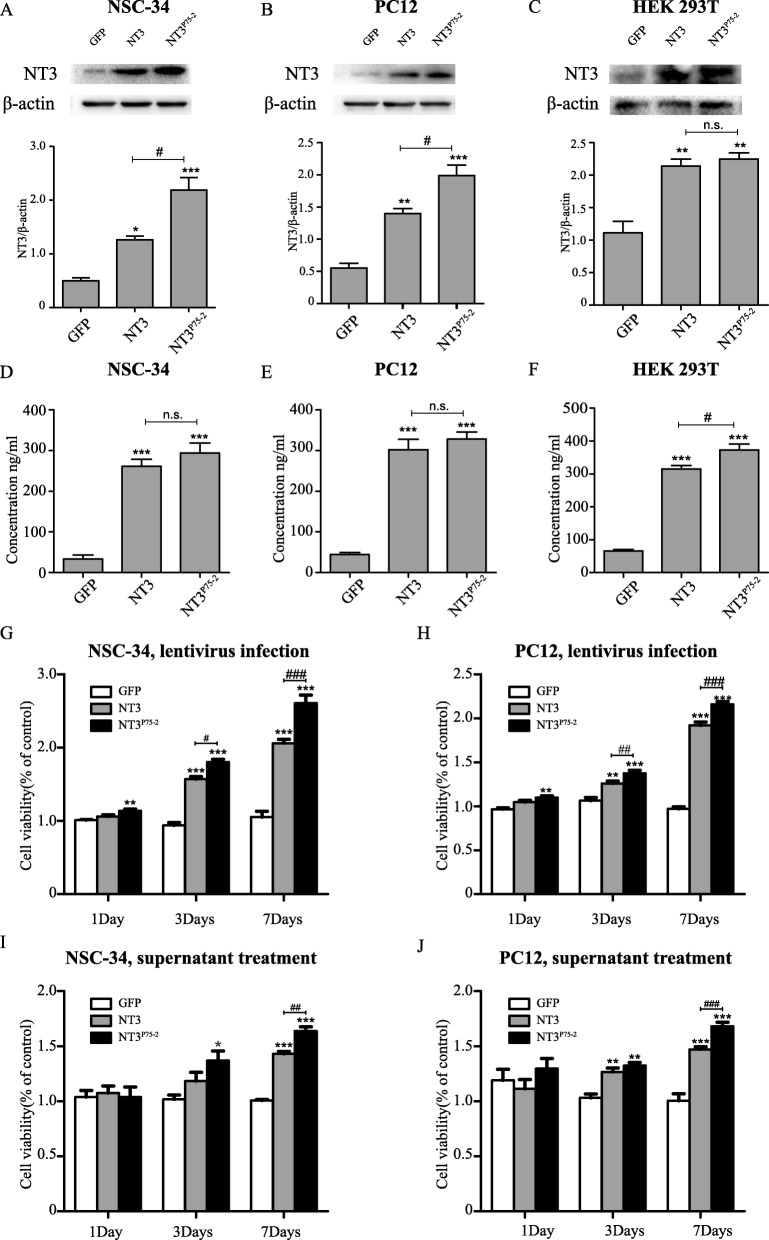
NT3^P75-2^ induction improves the cell growth rate of NSC-34 and PC12 cells in vitro. **a** NT3 expression levels of GFP, NT3, and NT3^P75-2^ in NSC-34 were analyzed by western blot, and the statistical data was also presented (^#^*P* < 0.05, *P < 0.05, ***P < 0.001 by one-way ANOVA followed by Bonferroni’s multiple comparison test, *n* = 3). **b** NT3 expression levels of GFP, NT3, and NT3^P75-2^ in PC12 were analyzed by western blot, and the statistical data was also presented (^#^*P* < 0.05, **P < 0.01, ***P < 0.001 by one-way ANOVA followed by Bonferroni’s multiple comparison test, *n* = 3). **c** NT3 expression levels of GFP, NT3, and NT3^P75-2^ in HEK293T were analyzed by western blot, and the statistical data was also presented (**P < 0.01 by one-way ANOVA followed by Bonferroni’s multiple comparison test; n.s., no significance, *n* = 3). **d** NT3 concentrations of cellular supernatant of GFP, NT3, and NT3^P75-2^-infected NSC-34 cells were assessed by ELISA (***P < 0.001 by one-way ANOVA followed by Bonferroni’s multiple comparison test; n.s., no significance, *n* = 6). **e** NT3 concentrations of cellular supernatant of GFP, NT3, and NT3^P75-2^-infected PC12 cells were assessed by ELISA (***P < 0.001 by one-way ANOVA followed by Bonferroni’s multiple comparison test; n.s., no significance, *n* = 6). **f** NT3 concentrations of cellular supernatant of GFP, NT3, and NT3^P75-2^-infected HEK293T cells were assessed by ELISA (^#^*P* < 0.05, ***P < 0.001 by one-way ANOVA followed by Bonferroni’s multiple comparison test, *n* = 6). **g** CCK8 assay measurement of cell growth of NSC-34 cells infected with lentiviruses of GFP, GFP-NT3, or GFP-NT3^P75-2^ (^#^*P* < 0.05, ^##^*P* < 0.01, ^###^*P* < 0.001, **P < 0.01, ***P < 0.001 by two-way ANOVA followed by Bonferroni’s multiple comparison test, *n* = 6). **h** CCK8 assay measurement of cell growth of PC12 cells infected with lentiviruses of GFP, GFP-NT3, or GFP-NT3^P75-2^ (^##^P < 0.01, ^###^*P* < 0.001, **P < 0.01, ***P < 0.001 by two-way ANOVA followed by Bonferroni’s multiple comparison test, *n* = 6). **i** CCK8 assay measurement of cell growth of NSC-34 cells after treatment with the supernatant of the HEK293T cells which were respectively infected with lentiviruses of GFP, NT3, or NT3^P75-2^ (^##^*P* < 0.01, *P < 0.05, ***P < 0.001 by two-way ANOVA followed by Bonferroni’s multiple comparison test, *n* = 6). **j** CCK8 assay measurement of cell growth of PC12 cells after treatment with the supernatant of the HEK293T cells which were respectively infected with lentiviruses of GFP, NT3, or NT3^P75-2^ (^###^*P* < 0.001, **P < 0.01, ***P < 0.001 by two-way ANOVA followed by Bonferroni’s multiple comparison test, *n* = 6)

NSC-34 and PC12 were infected with different lentiviruses; the overexpression of NT3 or NT3^P75-2^ in NSC-34, PC12, and HEK293T cell lines were confirmed by western blot (Fig. 1a–c). The secretion level of NT3 or NT3^P75-2^ was also detected and confirmed by ELISA in NSC-34, PC12, and HEK293T cell line after transfection with NT3 or NT3^P75-2^ plasmids (Fig. 1d–f).

CCK8 assay showed that overexpression of NT3 or NT3^P75-2^ could significantly enhance the cell growth rate of NSC-34 and PC12 cells at 3 and 7 days post-infection (Fig. 1g, h). In addition, this effect was higher in the NT3^P75-2^ group as compared to the NT3 group (Fig. 1g, h), which indicates that NT3^P75-2^ has enhanced effects for improving the cell growth rate of neuron character cell lines.

To further confirm this phenomenon, after the overexpression of NT3 or NT3^P75-2^, the supernatant of HEK293T cell lines was used for culturing NSC-34 or PC12 cells. The concentration of NT3 or NT3^P75-2^ was adjusted to 200 ng/ml using ELISA. It was observed that the supernatant of NT3 or NT3^P75-2^ could significantly improve the cell growth rate of NSC-34 and PC12 (Fig. 1i, j), which is consistent with NT3 or NT3^P75-2^ overexpression. Importantly, compared to NT3 supernatant, NSC-34 and PC12 treated with NT3^P75-2^ supernatant have a higher cell growth rate at 3 and 7 days after treatment (Fig. 1i, j). However, compared to supernatant treatment condition, lentivirus infection seems more efficient to promote cell growth rate (Additional file 3).

### NT3^P75-2^ overexpression enhances the growth of BMSCs in vitro

**Fig. 2:**
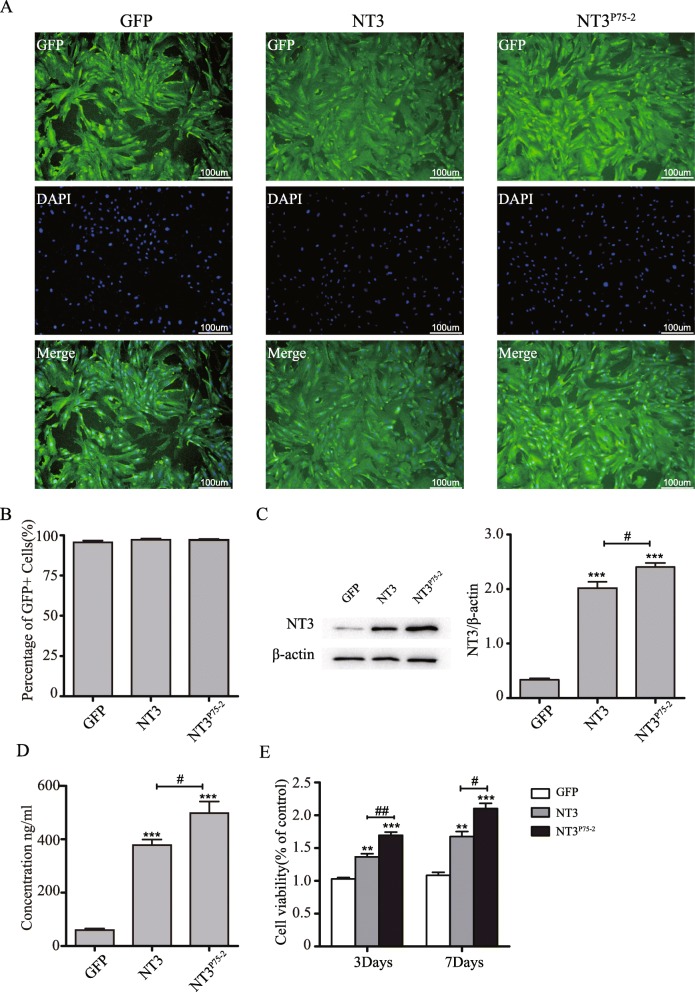
NT3^P75-2^ overexpression enhances the growth of BMSCs in vitro. **a** Representative images of green fluorescence protein (GFP) in BMSCs after infecting with lentiviruses (GFP, NT3, or NT3^P75-2^) at 3 days post-infection (*n* = 3). **b** The counting data of percent of GFP-positive BMSCs in different groups. **c** NT3 expression levels in BMSCs after infecting with lentiviruses (GFP, NT3, or NT3^P75-2^) were analyzed by western blot, and the statistical data was also presented (^#^*P* < 0.05, ***P < 0.001 by one-way ANOVA followed by Bonferroni’s multiple comparison test, *n* = 3). **d** NT3 concentrations of cellular supernatant in BMSCs after infecting with lentiviruses (GFP, NT3, and NT3^P75-2^) were assessed by ELISA (^#^*P* < 0.05, ***P < 0.001 by one-way ANOVA followed by Bonferroni’s multiple comparison test, *n* = 6). **e** The cell growth of BMSCs after infecting with lentiviruses (GFP, NT3, or NT3^P75-2^) was measured by CCK8 assays (^#^*P* < 0.05, ^###^*P* < 0.001, **P < 0.01, ***P < 0.001 by two-way ANOVA followed by Bonferroni’s multiple comparison test, *n* = 6)

BMSCs were infected with different lentiviruses (GFP, GFP-NT3, and GFP-NT3^P75-2^). The infection efficiency was confirmed by immunostaining of GFP at 3 days after infection (Fig. 2a). The results showed infection efficiencies of 95.6%, 97.3%, and 97.1%, respectively (Fig. 2b). In addition, the overexpression or secretion of NT3 or NT3^P75-2^ in BMSCs after infection were confirmed by western blot or ELISA (Fig. 2c, d). After infection of different lentiviruses (GFP, GFP-NT3, and GFP-NT3^P75-2^), both NT3 or NT3^P75-2^ significantly improved the cell growth rate of BMSCs with NT3^P75-2^ producing a much greater effect than NT3 at 3 and 7 days post-infection (Fig. 2e).

### NT3^P75-2^ overexpression induces TrkC pathway activation while reducing the activation of P75NTR signal in PC12 cells

**Fig. 3:**
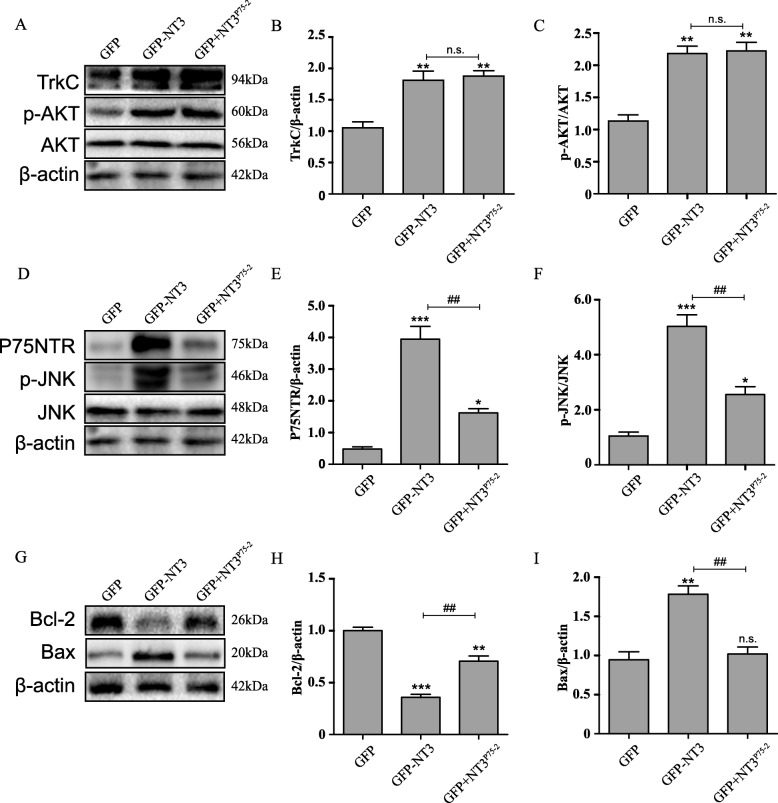
NT3^P**7**5-2^ overexpression induces TrkC pathway activation while reducing the activation of P75NTR signal in PC12 cells. **a**–**c** Western blot analysis of the protein levels of TrkC, AKT, p-AKT, and β-actin. Quantification of TrkC and p-AKT protein expression levels (**P < 0.01 by one-way ANOVA followed by Bonferroni’s multiple comparison test; n.s., no significance, *n* = 3). **d**–**f** Western blot analysis of the protein levels of P75NTR, JNK, p-JNK, and β-actin. Quantification of P75NTR and p-JNK protein expression levels (^##^*P* < 0.01, *P < 0.05, ***P < 0.001 by one-way ANOVA followed by Bonferroni’s multiple comparison test, *n* = 3). **g**–**i** Western blot analysis of the protein levels of Bcl-2, Bax, and β-actin. Quantification of Bcl-2 and Bax protein expression levels (^##^*P* < 0.01, **P < 0.01, ***P < 0.001 by one-way ANOVA followed by Bonferroni’s multiple comparison test; n.s., no significance, *n* = 3)

According to our hypothesis, the mutation of NT3 (NT3^P75-2^) could reduce the affinity of NT3 to P75NTR, and then influence the activation of the downstream JNK signal pathway, which plays a critical role in cell death. At 3 days post-infection of PC12 cells with the different lentiviruses, the PC12 cell sample was collected for western blot analysis. The mutation of NT3 (NT3^P75-2^) reduced the affinity of NT3 to P75NTR (Fig. 3a, b) but had no significant effect on TrkC activation (Fig. 3d, e). Activation of Akt, a downstream mediator of TrkC, promotes cell survival [30]; thus, we determined the effect of the lentiviruses on Akt activation. Akt protein levels were unchanged by the treatments. NT3 and NT3^P75-2^ increased phospho-Akt levels to a similar degree (Fig. 3a, c). In contrast, activation of JNK, a downstream mediator of P75NTR, induces cell death. Overexpression of NT3^P75-2^ reduced phospho-JNK protein, a downstream mediator of P75NTR which induces cell death, compared to NT3, while JNK was unaffected (Fig. 3d, f). Furthermore, the expression level of cell apoptosis markers (Bax and Bcl-2) was also determined which are the downstream mediators of JNK. The results showed that NT3 could significantly reduce Bcl-2 expression and induce Bax expression, while NT3^P75-2^ have no effect for Bax expression and have a weak effect for Bcl-2 expression (Fig. 3g–i), which are consistent with the weak JNK activation by NT3^P75-2^. In summary, the mutation of NT3 (NT3^P75-2^) improved the cell growth rate by reducing the activation of P75NTR and JNK signal (Additional file 2).

### Transplantation of NT3^P75-2^ gene-modified BMSCs promotes neurological function recovery in a mouse TBI model

**Fig. 4:**
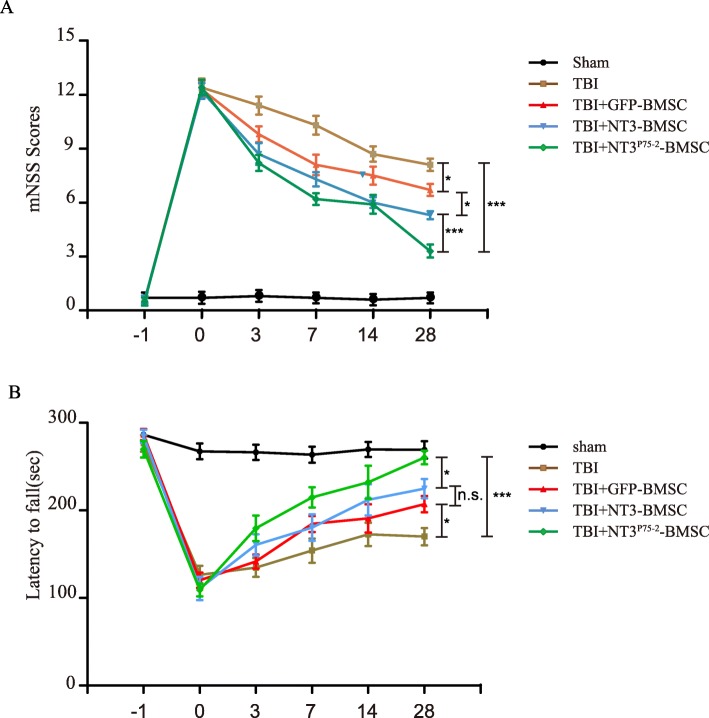
Transplantation of NT3^P75-2^ gene-modified BMSCs promotes neurological function recovery of the mouse TBI model. **a** The modified neurological severity scores of the mice in each group were tested at 3, 7, 14, and 28 days post-TBI (*P < 0.05, ***P < 0.001 by two-way ANOVA followed by Bonferroni’s multiple comparison test, *n* = 10). **b** Rotarod tests were performed to assess the motor abilities and coordination at 3, 7, 14, and 28 days post-TBI (*P < 0.05, ***P < 0.001 by two-way ANOVA followed by Bonferroni’s multiple comparison test; n.s.,no significance, *n* = 10)

The next step was to determine if the improved cell growth rate observed had any functional significance, that is, if it could enhance neurological functional recovery in a mouse TBI model. Transplantation of NT3 and NT3^P75-2^ gene-modified BMSCs significantly improved neurological function recovery as measured by the mNSS compared to the TBI+GFP-BMSC group at day 28 (Fig. 4a). Furthermore, a more significant recovery was observed with transplantation of NT3^P75-2^ gene-modified BMSCs as compared to NT3 gene-modified BMSCs as measured by the mNSS and rotarod test (Fig. 4a, b).

### Overexpression of NT3^P75-2^ promotes the survival of transplanted BMSCs after TBI

**Fig. 5:**
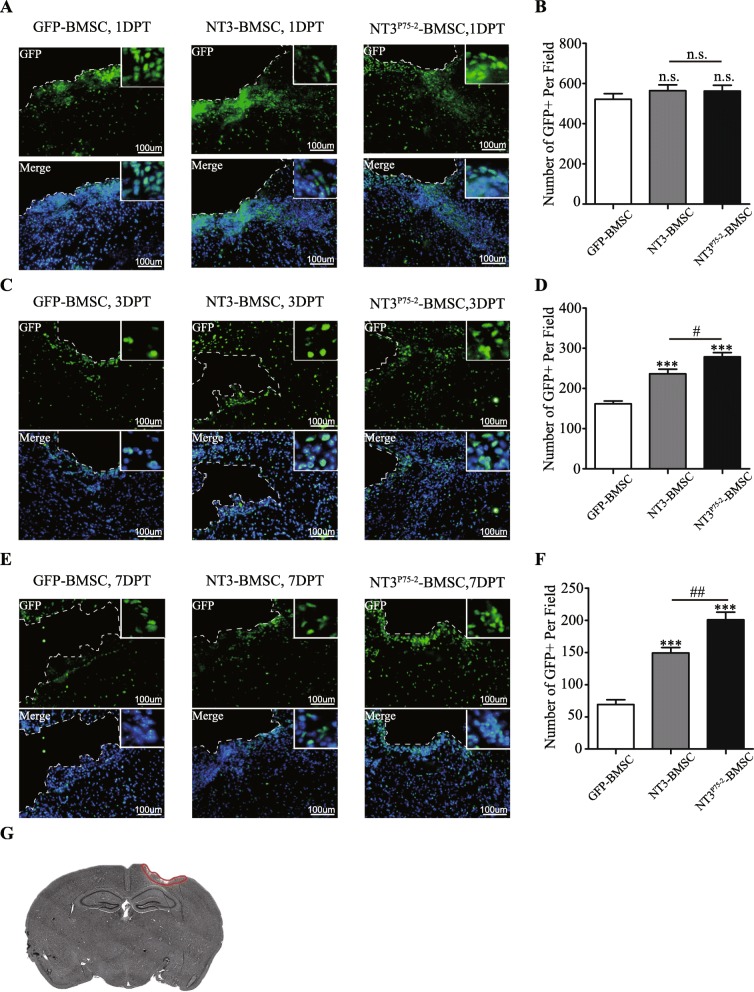
Overexpression of NT3^P75-2^ promotes the survival of transplanted BMSCs after TBI. **a** The representative images of GFP staining of BMSCs at 1 day after transplantation in different groups (GFP-BMSCs, NT3-BMSCs, NT3^P75-2^-BMSCs). The nuclei are stained by DAPI. The dotted line indicated the brain injury area (scale bar, 100 μm). **b** Quantification analysis of GFP-positive cells in each group (GFP-BMSCs, NT3-BMSCs, NT3^P75-2^-BMSCs) (n.s.,no significance, *n* = 4). **c** The representative images of GFP staining of BMSCs at 3 days after transplantation in different groups (GFP-BMSCs, NT3-BMSCs, NT3^P75-2^-BMSCs). The nuclei are stained by DAPI. The dotted line indicated the brain injury area (scale bar, 100 μm). **d** Quantification analysis of GFP-positive cells in each group (GFP-BMSCs, NT3-BMSCs, NT3^P75-2^-BMSCs) (^#^*P* < 0.05, ***P < 0.001 by one-way ANOVA followed by Bonferroni’s multiple comparison rest, *n* = 4). **e** The representative images of GFP staining of BMSCs at 7 days after transplantation in different groups (GFP-BMSCs, NT3-BMSCs, NT3^P75-2^-BMSCs). The nuclei are stained by DAPI. The dotted line indicated the brain injury area (scale bar, 100 μm). **f** Quantification analysis of GFP-positive cells in each group (GFP-BMSCs, NT3-BMSCs, NT3^P75-2^-BMSCs) (^##^*P* < 0.01, ***P < 0.001 by one-way ANOVA followed by Bonferroni’s multiple comparison test, *n* = 4). **g** The diagram indicates the area for image capture

After confirmation of the high infection efficiency (Fig. 2a, b), the gene-modified BMSCs were transplanted into the epicenter of the injury site at 1 day post-TBI. At 1, 3, or 7 days post-transplantation, the mice were sacrificed, and GFP staining was performed. GFP staining was used as a marker to determine cell survival. The results showed that NT3 overexpression improved BMSC survival compared to the GFP-BMSCs group at 3 or 7 days post-transplantation (Fig. 5c–f). Moreover, NT3^P75-2^ overexpression further enhanced the survival of BMSCs compared to the NT3-BMSCs group (Fig. 5c–f).

### NT3^P75-2^ overexpression inhibits glial cells activation after TBI

**Fig. 6:**
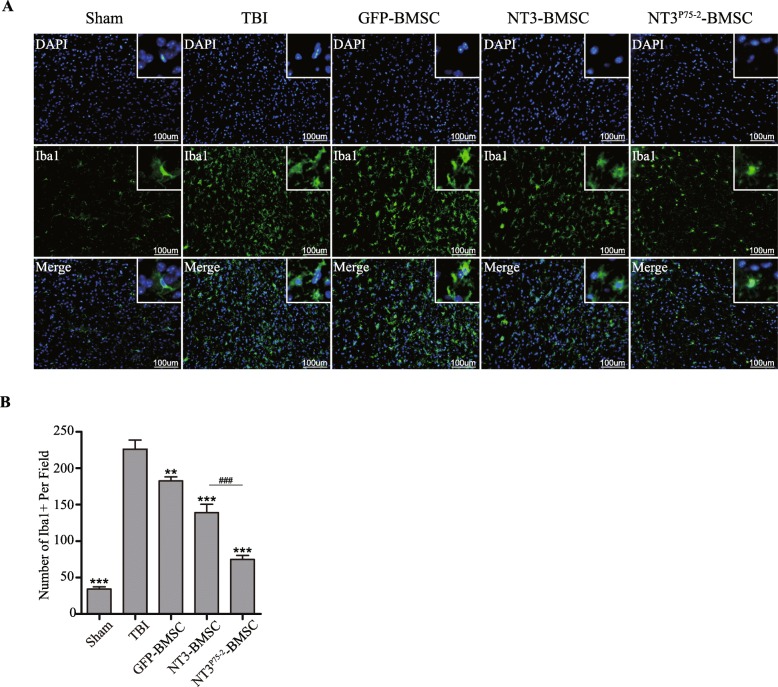
NT3^P75-2^ overexpression inhibits microglia activation after TBI. **a** Representative images of Iba1 staining in different groups (Sham, TBI, GFP-BMSCs, NT3-BMSCs, NT3^P75-2^-BMSCs) (scale bar, 100 μm). **b** Quantification analysis of Iba1-positive cells in each group (^###^*P* < 0.001, **P < 0.01, ***P < 0.001 by one-way ANOVA followed by Bonferroni’s multiple comparison test, *n* = 4)

Glial cell activation plays a critical role in functional recovery after TBI; thus, astrocyte and microglia activation was determined by GFAP and Iba1 staining, respectively, around the injury site after transplantation. At 7 days post-transplantation, the mice were sacrificed, and staining was performed. Both NT3 and NT3^P75-2^ gene-modified BMSCs significantly reduced Iba1-positive cell number around the injury site after TBI, with a more significant reduction observed in the NT3^P75-2^ gene-modified BMSCs group (Fig. 6a, b). GFAP staining was performed to evaluate the astrocyte activation state after gene-modified BMSC transplantation. GFAP-positive cell number was significantly decreased after transplantation of NT3 and NT3^P75-2^ gene-modified BMSCs after TBI (Additional file 1: Figure S1A and Additional file 1: Figure S1B). Importantly, NT3^P75-2^ gene-modified BMSC transplantation further reduced GFAP-positive cell numbers compared to the NT3-BMSCs group (Additional file 1: Figure S1A and Additional file 1: Figure S1B).

### NT3^P75-2^ overexpression reduces injury lesion volume and brain edema after TBI

**Fig. 7:**
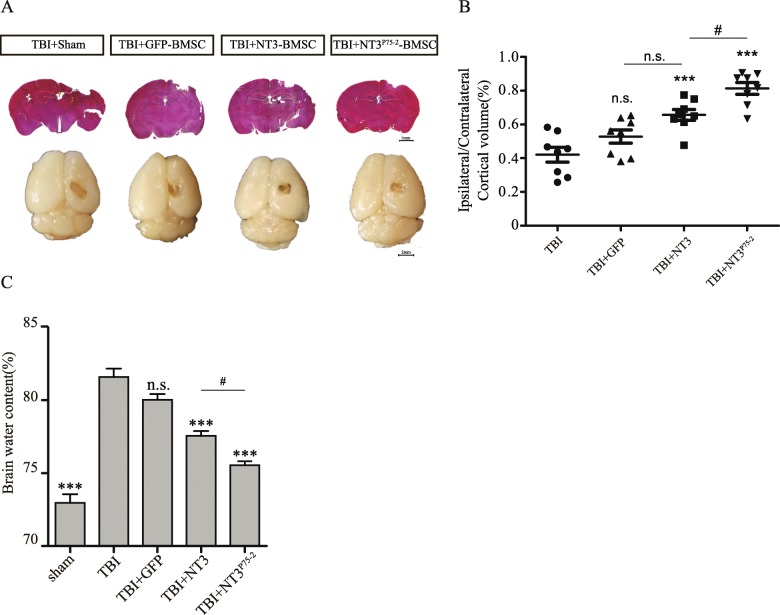
NT3^P75-2^ overexpression reduces brain lesion volume and brain edema after TBI. **a** The representative image of the whole mouse brain and brain sections of H&E staining at 7 days post-TBI with different treatments. **b** Quantification analysis of lesion volume in each group (n.s., no significance; ^#^*P* < 0.05, ***P < 0.001 by one-way ANOVA followed by Bonferroni’s multiple comparison test, *n* = 8). **c** Brain edema content analysis after TBI in different groups (n.s., no significance; ^#^*P* < 0.05, *P < 0.05, **P < 0.01, ***P < 0.001 by one-way ANOVA followed by Bonferroni’s multiple comparison test, *n* = 6)

To test whether NT3^P75-2^ gene-modified BMSC transplantation could reduce injury lesion volume after TBI, lesion volumes were measured. As predicted, compared to the GFP-BMSCs group, NT3 gene-modified BMSC transplantation significantly reduced the lesion volume after TBI (Fig. 7a, b). More importantly, NT3^P75-2^ gene-modified BMSC transplantation further reduced the injury volume as compared to the NT3-BMSCs treatment group (Fig. 7a, b). In addition, the brain edema level was also significantly reduced by NT3^P75-2^ gene-modified BMSC transplantation as compared to the NT3-BMSCs treatment group (Fig. 7c).

## Discussion

In this study, we firstly demonstrated that the transplantation of NT3^P75-2^ gene-modified BMSCs in the early stage of TBI could significantly improve the neurological functional recovery. Meanwhile, this therapeutic effect is of a higher magnitude when compared to the NT3 gene-modified BMSC transplantation treatment group. In addition, NT3^P75-2^ mutation had a reduced affinity with P75NTR compared to the NT3 group. This effect enhanced certain biological functions including increasing the cell growth rate of NSC-34, PC12, and BMSCs; promoting BMSC cell survival; decreasing glial cell activation; and reducing the injury lesion volume after TBI. Taken together, this specific gene mutation of NT3 in BMSCs could enhance their therapeutic effects as a novel bioengineering approach and may provide a new promising drug discovery strategy for improving the neurological functional recovery after TBI.

Neurotrophin 3 is a multifunctional neurotrophin, which can be secreted to the extracellular space [31]. In vivo, NT3 contributes to the survival of sensory and motor neurons, reduces the excitability of motor neurons, enhances the differentiation and plasticity of neuronal cells, promotes axon growth, and takes part in nerve repair post-damage [32, 33]. P75NTR is a member of the TNFR superfamily which can bind with all kinds of neurotrophins and induce cell death via apoptosis [34]. Hence, all neurotrophins bind to P75NTR with a similar affinity, while Trk receptors (with different subtypes) are specific binding sites for different neurotrophins with a high affinity. For instance, NGF binds to TrkA, BDNF binds to TrkB, and NT3 binds to TrkC receptor [35, 36]. Through binding to TrkC, NT3 promotes the survival of neurons under stimulation; however, binding to P75NTR can induce cell apoptosis [30, 37]. Previous studies show that altering the binding sites of these receptors could change the molecular biological functions of the neurotrophins [38]. Kusano and his colleagues found that one of the mutants, NT3/D15A, a neurotrophin with the abilities to bind TrkB and TrkC, could promote nerve regeneration and functional recovery after SCI [39]. Moreover, Sung et al. discovered that NGF^R100W^ have the capacity to bind to TrkA and stimulate its signaling pathway, therefore enhancing neuronal cell survival and differentiation, but the failure of activating P75NTR could significantly decrease the pain produced by NGF [40]. Consistently, our findings also provide evidence that NT3^P75-2^ overexpression enhances the cell growth rate of NSC-34, PC12, and BMSCs as well as reduces the affinity for P75NTR, as well as the downstream JNK signaling pathway. The mechanism of acute craniocerebral trauma is a series of complicated pathophysiological cascade reactions, such as the generation of reactive oxygen species, excitotoxicity, pyroptosis, neuronal apoptosis, inflammation, and diffuse axonal injury [41, 42]. The neuro-inflammation is considered as a leading cause of secondary insults after TBI [43]. In the early stages of acute brain injury, injured cells release a series of harmful signals to produce immune responses [44]. The immune responses then stimulate inflammatory reactive cells to secrete many kinds of cytokines and adhesion molecules [45]. Microglia, known as the macrophagocytes of the brain and spinal cord, are the first in response to the injury, followed by endothelial cells and astrocytes. In the NT3/TrkC system, NT3 plays an important role in neuroprotective processes. Dedoni’s group reported that NT3 is able to induce transphosphorylation of TrkC and activate the downstream signaling pathways, including Akt [31]. In the PI3K/Akt and MAPK signaling pathways, Akt2 and Akt3 are critical for the regulation of astrogliosis [46]. It has been previously shown that P75NTR is closely related to microglia activation, and TAT-Pep5, P75NTR-specific antagonists, can prevent the activation of microglia by P75NTR [47]. Interestingly, in our study, we demonstrated that NT3^P75-2^ could enhance the function of NT3 to inhibit microglia and astrocyte cell activation via reducing the binding to P75NTR. However, the detailed changes in the neuro-inflammatory environment after NT3P^75-2^ gene-modified BMSC transplantation in TBI mice remain to be fully elucidated.

In order to strengthen the argument for the role of stem cell transplantation therapy, an increasing number of studies focus on how to enhance the survival of transplanted stem cells. Recently, there are many preconditioning triggers that are utilized to promote the tolerance of grafted cells to different living environments [48–50]. For instance, the co-transplantion of BMSCs with NGF and Noggin could clearly enhance the survival of BMSCs compared to the transfected GFP alone group in the ischemic brain area [51]. NT3^P75-2^ enhances grafted survival of Schwann cells and axon growth after spinal cord injury in rats via reducing affinity to P75NTR [20]. Overexpression of Hif-1α promotes the survival of BMSCs by activating the AMPK and mTOR signaling pathways, as well as by regulating autophagy [52]. Daviaud and his colleagues indicated that adherence to NT3-releasing pharmacologically active microcarriers (PAM) may contribute to the survival, differentiation, and neuroprotective function in an in vivo model of PD [53]. Overexpression of vascular endothelial growth factor (VEGF) promoted transplantation of VEGF-BMSCs to improve ischemic neurological outcome in a middle cerebral artery occlusion (MCAO) rat model [54–56]. In the same light, our results suggest that overexpression of NT3^P75-2^ improves the survival of BMSCs in the injured region of the mouse TBI model, which may partially explain how NT3^P75-2^ gene-modified BMSC transplantation could improve neurological function recovery of TBI.

TBI leads to the damage to the lesion cortex, as well as cellular and molecular dysfunction in the primary lesion, which eventually results in praxiological impairments [57]. After TBI or ischemic stroke, the mNSS tests and rotarod are commonly utilized to estimate motor, coordination, and neurological function deficits [58–60]. Intravenous injection of NT3 has been shown to effectively enhance both motor and sensory function recovery after stroke, compared to the vehicle sham group [61]. But, in terms of our existing knowledge, NT3^P75-2^ combined with BMSC therapy has not been investigated in TBI. Based on the data we presented in this study, we found that transplantation of NT3^P75-2^ gene-modified BMSCs could reduce at least a 30% lesion volume compared to the BMSCs only group and 10% compared to the NT3-BMSCs group at 7 days post-transplantation. In addition, transplantation of NT3^P75-2^ gene-modified BMSCs at an early-stage of TBI could dramatically improve the functional recovery after TBI. Even though there are neurological protection effects caused by the NT3^P75-2^ gene-modified BMSC transplantation, the potential effects of NT3^P75-2^ therapy only may still need to be further explored.

In conclusion, based on this study, we found that NT3^P75-2^ greatly improves neurological functional recovery after TBI. The potential underlying mechanism includes the improvement of BMSC survival, the reduction of glial cell activation, and the alleviation of injury lesion volumes after TBI. Although the application of NT3^P75-2^ shows great promise for TBI therapy, the potential side effects and further molecular mechanisms still need to be broadly explored.

## Supplementary information

**Additional file 1:** **Figure S1.** NT3P75-2 overexpression could inhibit astrocyte activation after TBI. (A) Representative images of GFAP staining in different groups (Sham, TBI, GFP-BMSCs, NT3-BMSCs, NT3P75-2-BMSCs).(Scale Bar, 100 um) (B) Quantification analysis of GFAP positive cells in each groups (n.s. no significance, ###*P*<0.001, ***P<0.001 by one-way ANOVA followed by Bonferroni’s Multiple Comparison Test, *n* = 4).

**Additional file 2:** **Figure S2.** NT3P75-2 overexpression induces TrkC expression, while reducing the P75NTR expression. (A) The relative mRNA expression level of TrkC at 3 days post infection. (n.s. no significance, **P<0.01 by one-way. ANOVA followed by Bonferroni’s Multiple Comparison Test, n = 4). (B) The relative mRNA expression level of P75NTR at 3 days post infection. (###P<0.001, ***P<0.001 by one-way. ANOVA followed by Bonferroni’s Multiple Comparison Test, n = 4).

**Additional file 3:** **Figure S3.** NT3P75-2 induction improves cell growth of PC12 cells in vitro. (A) NT3 expression levels of GFP, NT3 and NT3P75-2 in PC12 were analyzed by Western blot. And the statistical data was also presented. (**P<0.01 by one-way ANOVA followed by Bonferroni’s Multiple Comparison Test, n.s., no significance, *n*=3). (B) CCK8 assays measurement of cell growth of PC12 cells infected with lentiviruses of GFP, GFP-NT3 or GFP-NT3P75-2.(#*P*<0.05, ##P<0.01, **P<0.01, ***P<0.001 by two-way ANOVA followed by Bonferroni’s Multiple Comparison Test, *n*=6).

## Data Availability

All data generated or analyzed during this study are included in the published article.

## Abbreviations

akt: Protein kinase B
ANOVA: Analysis of variance
BDNF: Brain-derived neurotrophic factor
BMSCs: Bone marrow-derived mesenchymal stem cells
CCI: Controlled cortical impact
CCK8: Cell Counting Kit-8
DMEM: Dulbecco’s modified Eagles medium
DMSO: Dimethylsulfoxide
DPT: Days post-transplantation
ELISA: Enzyme-linked immunosorbent assay
FBS: Fetal bovine serum
GDNF: Glial cell line-derived neurotrophic factor
GFAP: Glial fibrillary acidic protein
GFP: Green fluorescent protein
Iba1: Ionized calcium-binding adaptor molecule 1
JNK: c-Jun N-terminal kinase
MCAO: Middle cerebral artery occlusion
mNSS: Modified neurological severity score
NGF: Nerve growth factor
NT3: Neurotrophin 3
P75NTR: P75 neurotrophin receptor
PBS: Phosphate-buffered saline
PD: Parkinson’s disease
PFA: Paraformaldehyde
PVDF: Polyvinylidene difluoride
RIPA: Radioimmunoprecipitation assay
SCI: Spinal cord injury
SEM: Scanning electron microscope
TBI: Traumatic brain injury
TNFR: Tumor necrosis factor receptor
TrK: Receptor tyrosine kinase
TrkC: Neurotrophic tyrosine kinase, receptor, type 3
VEGF: Vascular endothelial growth factor

## References

[1] Albayram O, Kondo A, Mannix R, Smith C, Tsai CY, Li C, Herbert MK, Qiu J, Monuteaux M, Driver J, Yan S, Gormley W, Puccio AM, Okonkwo DO, Lucke-Wold B, Bailes J, Meehan W, Zeidel M, Lu KP, Zhou XZ (2017). Cis P-tau is induced in clinical and preclinical brain injury and contributes to post-injury sequelae. Nat Commun.

[2] G.B.D.T.B. Injury, C. Spinal Cord Injury (2019). Global, regional, and national burden of traumatic brain injury and spinal cord injury, 1990–2016: a systematic analysis for the Global Burden of Disease Study 2016. Lancet Neurol.

[3] G.B.D. DALYs, H. Collaborators (2017). Global, regional, and national disability-adjusted life-years (DALYs) for 333 diseases and injuries and healthy life expectancy (HALE) for 195 countries and territories, 1990-2016: a systematic analysis for the Global Burden of Disease Study 2016. Lancet.

[4] Taylor CA, Bell JM, Breiding MJ, Xu L (2017). Traumatic brain injury-related emergency department visits, hospitalizations, and deaths - United States, 2007 and 2013. MMWR Surveill Summ.

[5] Xiong Y, Mahmood A, Meng Y, Zhang Y, Zhang ZG, Morris DC, Chopp M (2012). Neuroprotective and neurorestorative effects of thymosin beta4 treatment following experimental traumatic brain injury. Ann N Y Acad Sci.

[6] Borlongan CV, Glover LE, Tajiri N, Kaneko Y, Freeman TB (2011). The great migration of bone marrow-derived stem cells toward the ischemic brain: therapeutic implications for stroke and other neurological disorders. Prog Neurobiol.

[7] Nichols JE, Niles JA, DeWitt D, Prough D, Parsley M, Vega S, Cantu A, Lee E, Cortiella J (2013). Neurogenic and neuro-protective potential of a novel subpopulation of peripheral blood-derived CD133+ ABCG2+CXCR4+ mesenchymal stem cells: development of autologous cell-based therapeutics for traumatic brain injury. Stem Cell Res Ther.

[8] Li Y, Chopp M (2009). Marrow stromal cell transplantation in stroke and traumatic brain injury. Neurosci Lett.

[9] Feng Y, Ju Y, Cui J, Wang L (2017). Bone marrow stromal cells promote neuromotor functional recovery, via upregulation of neurotrophic factors and synapse proteins following traumatic brain injury in rats. Mol Med Rep.

[10] Shen Q, Yin Y, Xia QJ, Lin N, Wang YC, Liu J, Wang HP, Lim A, Wang TH (2016). Bone marrow stromal cells promote neuronal restoration in rats with traumatic brain injury: involvement of GDNF regulating BAD and BAX signaling. Cell Physiol Biochem.

[11] Lv B, Li F, Fang J, Xu L, Sun C, Han J, Hua T, Zhang Z, Feng Z, Wang Q, Jiang X (2016). Activated microglia induce bone marrow mesenchymal stem cells to produce glial cell-derived neurotrophic factor and protect neurons against oxygen-glucose deprivation injury. Front Cell Neurosci.

[12] Maisonpierre PC, Belluscio L, Squinto S, Ip NY, Furth ME, Lindsay RM, Yancopoulos GD (1990). Neurotrophin-3: a neurotrophic factor related to NGF and BDNF. Science.

[13] Blondy S, Christou N, David V, Verdier M, Jauberteau MO, Mathonnet M, Perraud A (2019). Neurotrophins and their involvement in digestive cancers. Cell Death Dis.

[14] Dechant G, Barde YA (2002). The neurotrophin receptor p75(NTR): novel functions and implications for diseases of the nervous system. Nat Neurosci.

[15] Teng KK, Felice S, Kim T, Hempstead BL (2010). Understanding proneurotrophin actions: recent advances and challenges. Dev Neurobiol.

[16] Underwood CK, Coulson EJ (2008). The p75 neurotrophin receptor. Int J Biochem Cell Biol.

[17] Hempstead BL (2002). The many faces of p75NTR. Curr Opin Neurobiol.

[18] Rabizadeh S, Oh J, Zhong LT, Yang J, Bitler CM, Butcher LL, Bredesen DE (1993). Induction of apoptosis by the low-affinity NGF receptor. Science.

[19] Scott AL, Ramer MS (2010). Schwann cell p75NTR prevents spontaneous sensory reinnervation of the adult spinal cord. Brain.

[20] Enomoto M, Bunge MB, Tsoulfas P (2013). A multifunctional neurotrophin with reduced affinity to p75NTR enhances transplanted Schwann cell survival and axon growth after spinal cord injury. Exp Neurol.

[21] Hu J, Chen L, Huang X, Wu K, Ding S, Wang W, Wang B, Smith C, Ren C, Ni H, ZhuGe Q, Yang J (2019). Calpain inhibitor MDL28170 improves the transplantation-mediated therapeutic effect of bone marrow-derived mesenchymal stem cells following traumatic brain injury. Stem Cell Res Ther.

[22] Smith DK, Yang J, Liu ML, Zhang CL (2016). Small molecules modulate chromatin accessibility to promote NEUROG2-mediated fibroblast-to-neuron reprogramming. Stem Cell Rep.

[23] Islam MM, Smith DK, Niu W, Fang S, Iqbal N, Sun G, Shi Y, Zhang CL (2015). Enhancer analysis unveils genetic interactions between TLX and SOX2 in neural stem cells and in vivo reprogramming. Stem Cell Rep.

[24] Niu W, Zang T, Zou Y, Fang S, Smith DK, Bachoo R, Zhang CL (2013). In vivo reprogramming of astrocytes to neuroblasts in the adult brain. Nat Cell Biol.

[25] Sarkar C, Jones JW, Hegdekar N, Thayer JA, Kumar A, Faden AI, Kane MA, Lipinski MM. PLA2G4A/cPLA2-mediated lysosomal membrane damage leads to inhibition of autophagy and neurodegeneration after brain trauma. Autophagy. 2019;1–20. [Epub ahead of print] 10.1080/15548627.2019.1628538 PMC6999646 31238788

[26] Xu X, Gao W, Cheng S, Yin D, Li F, Wu Y, Sun D, Zhou S, Wang D, Zhang Y, Jiang R, Zhang J (2017). Anti-inflammatory and immunomodulatory mechanisms of atorvastatin in a murine model of traumatic brain injury. J Neuroinflammation.

[27] Wu P, Zhao H, Gou X, Wu X, Zhang S, Deng G, Chen Q (2019). Targeted delivery of polypeptide nanoparticle for treatment of traumatic brain injury. Int J Nanomedicine.

[28] Lu Q, Gao L, Huang L, Ruan L, Yang J, Huang W, Li Z, Zhang Y, Jin K, Zhuge Q (2014). Inhibition of mammalian target of rapamycin improves neurobehavioral deficit and modulates immune response after intracerebral hemorrhage in rat. J Neuroinflammation.

[29] Yang J, Ding S, Huang W, Hu J, Huang S, Zhang Y, Zhuge Q (2016). Interleukin-4 ameliorates the functional recovery of Intracerebral hemorrhage through the alternative activation of microglia/macrophage. Front Neurosci.

[30] Dedoni S, Olianas MC, Ingianni A, Onali P (2017). Interferon-beta inhibits neurotrophin 3 signalling and pro-survival activity by upregulating the expression of truncated TrkC-T1 receptor. Mol Neurobiol.

[31] Ernfors P, Lee KF, Jaenisch R (1994). Mice lacking brain-derived neurotrophic factor develop with sensory deficits. Nature.

[32] Keefe Kathleen, Sheikh Imran, Smith George (2017). Targeting Neurotrophins to Specific Populations of Neurons: NGF, BDNF, and NT-3 and Their Relevance for Treatment of Spinal Cord Injury. International Journal of Molecular Sciences.

[33] Kathe C, Hutson TH, McMahon SB, Moon LD. Intramuscular neurotrophin-3 normalizes low threshold spinal reflexes, reduces spasms and improves mobility after bilateral corticospinal tract injury in rats. Elife. 2016;5. 10.7554/eLife.18146 PMC5070949 27759565

[34] Elshaer SL, Alwhaibi A, Mohamed R, Lemtalsi T, Coucha M, Longo FM, El-Remessy AB (2019). Modulation of the p75 neurotrophin receptor using LM11A-31 prevents diabetes-induced retinal vascular permeability in mice via inhibition of inflammation and the RhoA kinase pathway. Diabetologia.

[35] He XL, Garcia KC (2004). Structure of nerve growth factor complexed with the shared neurotrophin receptor p75. Science.

[36] Ultsch MH, Wiesmann C, Simmons LC, Henrich J, Yang M, Reilly D, Bass SH, de Vos AM (1999). Crystal structures of the neurotrophin-binding domain of TrkA, TrkB and TrkC. J Mol Biol.

[37] Hickman FE, Stanley EM, Carter BD (2018). Neurotrophin responsiveness of sympathetic neurons is regulated by rapid mobilization of the p75 receptor to the cell surface through TrkA activation of Arf6. J Neurosci.

[38] Malerba F, Paoletti F, Bruni Ercole B, Materazzi S, Nassini R, Coppi E, Patacchini R, Capsoni S, Lamba D, Cattaneo A (2015). Functional characterization of human ProNGF and NGF mutants: identification of NGF P61SR100E as a “painless” lead investigational candidate for therapeutic applications. PLoS One.

[39] Kusano K, Enomoto M, Hirai T, Tsoulfas P, Sotome S, Shinomiya K, Okawa A (2010). Transplanted neural progenitor cells expressing mutant NT3 promote myelination and partial hindlimb recovery in the chronic phase after spinal cord injury. Biochem Biophys Res Commun.

[40] Sung K, Ferrari LF, Yang W, Chung C, Zhao X, Gu Y, Lin S, Zhang K, Cui B, Pearn ML, Maloney MT, Mobley WC, Levine JD, Wu C (2018). Swedish nerve growth factor mutation (NGF(R100W)) defines a role for TrkA and p75(NTR) in nociception. J Neurosci.

[41] Liu W, Chen Y, Meng J, Wu M, Bi F, Chang C, Li H, Zhang L (2018). Ablation of caspase-1 protects against TBI-induced pyroptosis in vitro and in vivo. J Neuroinflammation.

[42] Kim H, Yu T, Cam-Etoz B, van Groen T, Hubbard WJ, Chaudry IH (2017). Treatment of traumatic brain injury with 17alpha-ethinylestradiol-3-sulfate in a rat model. J Neurosurg.

[43] Shi K, Zhang J, Dong JF, Shi FD (2019). Dissemination of brain inflammation in traumatic brain injury. Cell Mol Immunol.

[44] Roth TL, Nayak D, Atanasijevic T, Koretsky AP, Latour LL, McGavern DB (2014). Transcranial amelioration of inflammation and cell death after brain injury. Nature.

[45] Abdul-Muneer PM, Chandra N, Haorah J (2015). Interactions of oxidative stress and neurovascular inflammation in the pathogenesis of traumatic brain injury. Mol Neurobiol.

[46] Endersby R, Zhu X, Hay N, Ellison DW, Baker SJ (2011). Nonredundant functions for Akt isoforms in astrocyte growth and gliomagenesis in an orthotopic transplantation model. Cancer Res.

[47] Xu Z, Shi WH, Xu LB, Shao MF, Chen ZP, Zhu GC, Hou Q (2019). Resident microglia activate before peripheral monocyte infiltration and p75NTR blockade reduces microglial activation and early brain injury after subarachnoid hemorrhage. ACS Chem Neurosci.

[48] Yan F, Yao Y, Chen L, Li Y, Sheng Z, Ma G (2012). Hypoxic preconditioning improves survival of cardiac progenitor cells: role of stromal cell derived factor-1alpha-CXCR4 axis. PLoS One.

[49] Sakata H, Niizuma K, Yoshioka H, Kim GS, Jung JE, Katsu M, Narasimhan P, Maier CM, Nishiyama Y, Chan PH (2012). Minocycline-preconditioned neural stem cells enhance neuroprotection after ischemic stroke in rats. J Neurosci.

[50] Ren C, Li N, Li S, Han R, Huang Q, Hu J, Jin K, Ji X (2018). Limb ischemic conditioning improved cognitive deficits via eNOS-dependent augmentation of angiogenesis after chronic cerebral hypoperfusion in rats. Aging Dis.

[51] Ding J, Cheng Y, Gao S, Chen J (2011). Effects of nerve growth factor and Noggin-modified bone marrow stromal cells on stroke in rats. J Neurosci Res.

[52] Lv B, Li F, Han J, Fang J, Xu L, Sun C, Hua T, Zhang Z, Feng Z, Jiang X (2017). Hif-1alpha overexpression improves transplanted bone mesenchymal stem cells survival in rat MCAO stroke model. Front Mol Neurosci.

[53] Daviaud N, Garbayo E, Sindji L, Martinez-Serrano A, Schiller PC, Montero-Menei CN (2015). Survival, differentiation, and neuroprotective mechanisms of human stem cells complexed with neurotrophin-3-releasing pharmacologically active microcarriers in an ex vivo model of Parkinson’s disease. Stem Cells Transl Med.

[54] Zong X, Wu S, Li F, Lv L, Han D, Zhao N, Yan X, Hu S, Xu T (2017). Transplantation of VEGF-mediated bone marrow mesenchymal stem cells promotes functional improvement in a rat acute cerebral infarction model. Brain Res.

[55] Ren C, Yao Y, Han R, Huang Q, Li H, Wang B, Li S, Li M, Mao Y, Mao X, Xie L, Zhou L, Hu J, Ji X, Jin K (2018). Cerebral ischemia induces angiogenesis in the peri-infarct regions via Notch1 signaling activation. Exp Neurol.

[56] Xu X, Wang B, Ren C, Hu J, Greenberg DA, Chen T, Xie L, Jin K (2017). Recent progress in vascular aging: mechanisms and its role in age-related diseases. Aging Dis.

[57] Chen C, Zhong X, Smith DK, Tai W, Yang J, Zou Y, Wang LL, Sun J, Qin S, Zhang CL (2019). Astrocyte-specific deletion of Sox2 promotes functional recovery after traumatic brain injury. Cereb Cortex.

[58] Gao C, Qian Y, Huang J, Wang D, Su W, Wang P, Guo L, Quan W, An S, Zhang J, Jiang R (2017). A three-day consecutive fingolimod administration improves neurological functions and modulates multiple immune responses of CCI mice. Mol Neurobiol.

[59] He WM, Ying-Fu L, Wang H, Peng YP (2019). Delayed treatment of alpha5 GABAA receptor inverse agonist improves functional recovery by enhancing neurogenesis after cerebral ischemia-reperfusion injury in rat MCAO model. Sci Rep.

[60] Xu X, Wang B, Ren C, Hu J, Greenberg DA, Chen T, Xie L, Jin K (2017). Age-related impairment of vascular structure and functions. Aging Dis.

[61] Duricki DA, Drndarski S, Bernanos M, Wood T, Bosch K, Chen Q, Shine HD, Simmons C, Williams SCR, McMahon SB, Begley DJ, Cash D, Moon LDF (2019). Stroke recovery in rats after 24-hour-delayed intramuscular neurotrophin-3 infusion. Ann Neurol.

