# Supplementary material for: NT3P75-2 gene-modified bone mesenchymal stem cells improve neurological function recovery in mouse TBI model
Source: Stem Cell Res Ther. 2019 Oct 24;10:311. doi: 10.1186/s13287-019-1428-1 (PMC6814101; doi:10.1186/s13287-019-1428-1)
Supplement: Supplementary file 1 — Additional file 1: Figure S1. NT3P75-2 overexpression could inhibit astrocyte activation after TBI. (A) Representative images of GFAP staining in different groups (Sham, TBI, GFP-BMSCs, NT3-BMSCs, NT3P75-2-BMSCs).(Scale Bar, 100 um) (B) Quantification analysis of GFAP positive cells in each groups (n.s. no significance, ###P<0.001, ***P<0.001 by one-way ANOVA followed by Bonferroni’s Multiple Comparison Test, n = 4). [file 13287_2019_1428_MOESM1_ESM.pdf]

Supplement Figure 1

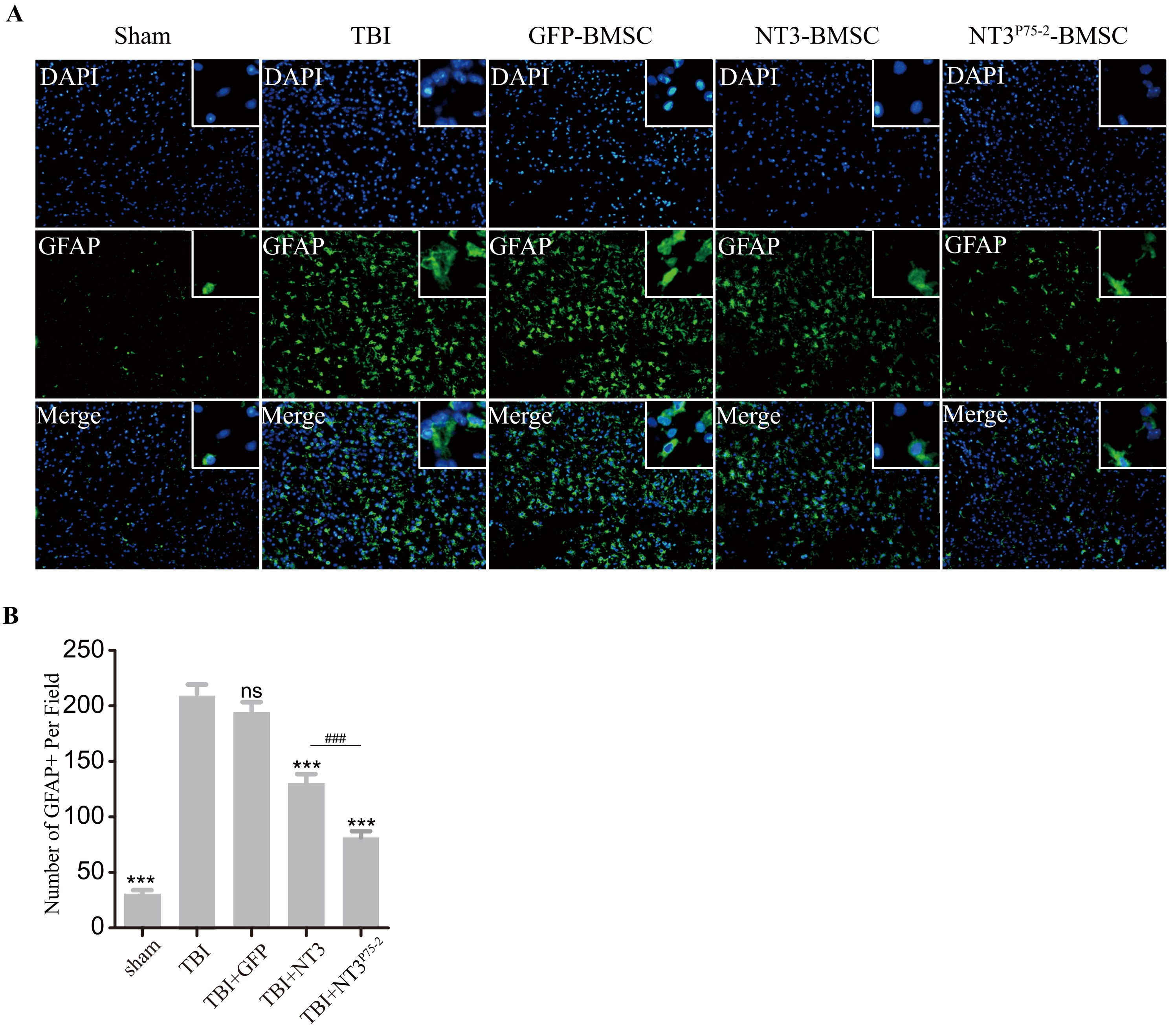

Supplement Figure 1. NT3P75-2 overexpression could inhibit astrocyte activation after TBI.
(A)Representative images of GFAP staining in different groups (Sham, TBI, GFP-BMSCs, NT3-BMSCs, NT3P75-2-BMSCs). (Scale Bar, 100 um)
(B)Quantification analysis of GFAP positive cells in each groups (n.s. no significance, ###P<0.001, ***P<0.001 by one-way ANOVA followed by Bonferroni's Multiple Comparison Test, n = 4).
