# Supplementary material for: NT3P75-2 gene-modified bone mesenchymal stem cells improve neurological function recovery in mouse TBI model
Source: Stem Cell Res Ther. 2019 Oct 24;10:311. doi: 10.1186/s13287-019-1428-1 (PMC6814101; doi:10.1186/s13287-019-1428-1)
Supplement: Supplementary file 2 — Additional file 2: Figure S2. NT3P75-2 overexpression induces TrkC expression, while reducing the P75NTR expression. (A) The relative mRNA expression level of TrkC at 3 days post infection. (n.s. no significance, **P<0.01 by one-way. ANOVA followed by Bonferroni’s Multiple Comparison Test, n = 4). (B) The relative mRNA expression level of P75NTR at 3 days post infection. (###P<0.001, ***P<0.001 by one-way. ANOVA followed by Bonferroni’s Multiple Comparison Test, n = 4). [file 13287_2019_1428_MOESM2_ESM.pdf]

Supplement Figure 2

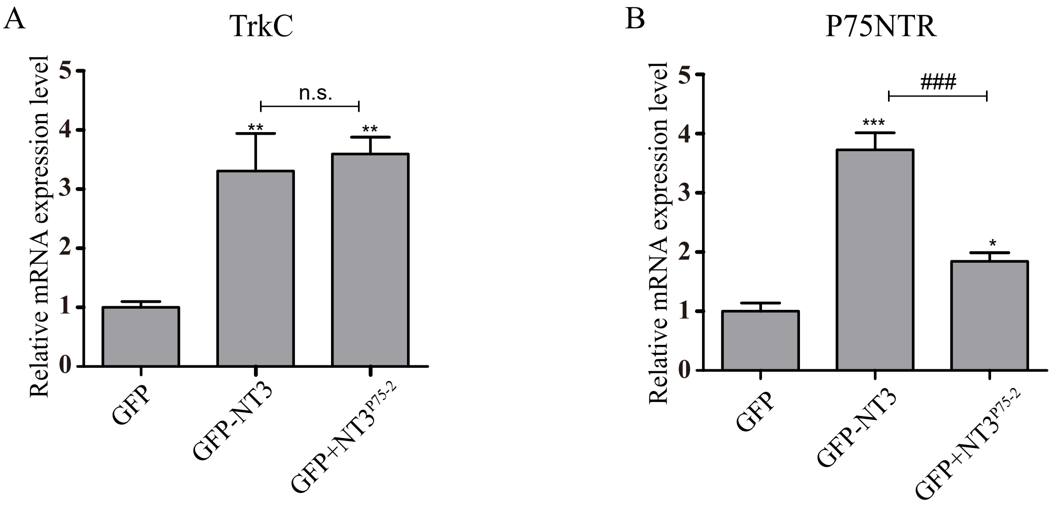

Supplement Figure 2. NT3P75-2 overexpression induces TrkC expression, while reducing the P75NTR expression.
(A) The relative mRNA expression level of TrkC at 3 days post infection. (n.s. no significance, **P<0.01 by one-way ANOVA followed by Bonferroni's Multiple Comparison Test, n = 4).
(B) The relative mRNA expression level of P75NTR at 3 days post infection. (###P<0.001, ***P<0.001 by one-way ANOVA followed by Bonferroni's Multiple Comparison Test, n = 4).
