# Supplementary material for: NT3P75-2 gene-modified bone mesenchymal stem cells improve neurological function recovery in mouse TBI model
Source: Stem Cell Res Ther. 2019 Oct 24;10:311. doi: 10.1186/s13287-019-1428-1 (PMC6814101; doi:10.1186/s13287-019-1428-1)
Supplement: Supplementary file 3 — Additional file 3: Figure S3. NT3P75-2 induction improves cell growth of PC12 cells in vitro. (A) NT3 expression levels of GFP, NT3 and NT3P75-2 in PC12 were analyzed by Western blot. And the statistical data was also presented. (**P<0.01 by one-way ANOVA followed by Bonferroni’s Multiple Comparison Test, n.s., no significance, n=3). (B) CCK8 assays measurement of cell growth of PC12 cells infected with lentiviruses of GFP, GFP-NT3 or GFP-NT3P75-2.(#P<0.05, ##P<0.01, **P<0.01, ***P<0.001 by two-way ANOVA followed by Bonferroni’s Multiple Comparison Test, n=6). [file 13287_2019_1428_MOESM3_ESM.pdf]

Supplement Figure 3

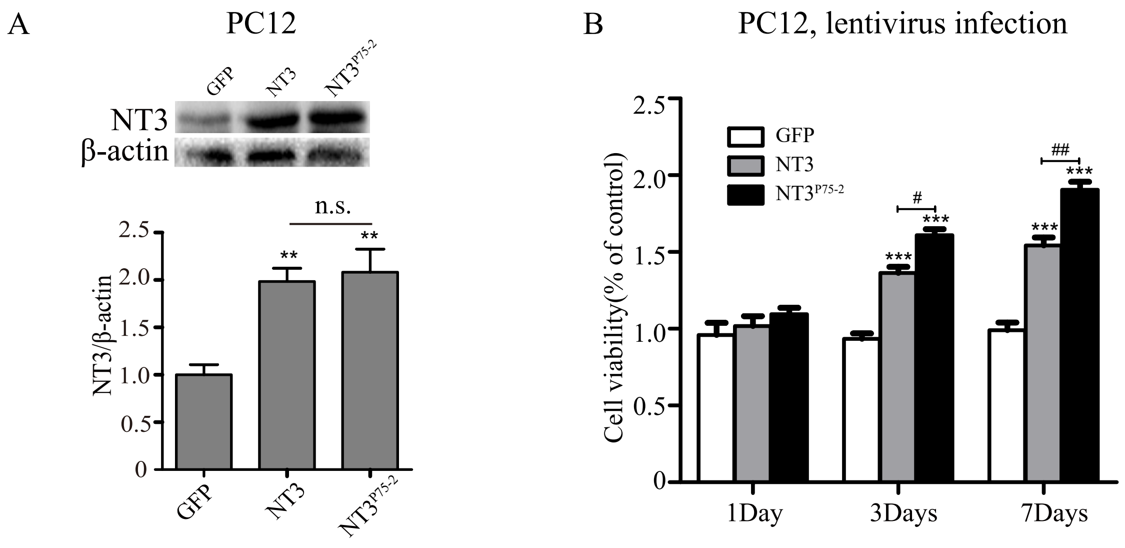

Supplement Figure 3. NT3P75-2 induction improves cell growth of PC12 cells in vitro.
(A) NT3 expression levels of GFP, NT3 and NT3P75-2 in PC12 were analyzed by Western blot. And the statistical data was also presented. (**P<0.01 by one-way ANOVA followed by Bonferroni's Multiple Comparison Test, n.s., no significance, n=3).
(B) CCK8 assays measurement of cell growth of PC12 cells infected with lentiviruses of GFP, GFP-NT3 or GFP-NT3P75-2. (#P<0.05, ##P<0.01, **P<0.01, ***P<0.001 by two-way ANOVA followed by Bonferroni's Multiple Comparison Test, n=6).
